# Influence of Amino Acid Feeding on Production of Calcimycin and Analogs in *Streptomyces chartreusis*

**DOI:** 10.3390/ijerph18168740

**Published:** 2021-08-19

**Authors:** Kirstin I. Arend, Julia E. Bandow

**Affiliations:** Applied Microbiology, Faculty of Biology and Biotechnology, Ruhr University Bochum, 44780 Bochum, Germany; kirstin.arend@rub.de

**Keywords:** metabolomics, specialized metabolite production, metabolic network

## Abstract

*Streptomyces chartreusis* NRRL 3882 produces the polyether ionophore calcimycin and a variety of analogs, which originate from the same biosynthetic gene cluster. The role of calcimycin and its analogs for the producer is unknown, but calcimycin has strong antibacterial activity. Feeding experiments were performed in chemically defined medium systematically supplemented with proteinogenic amino acids to analyze their individual effects on calcimycin synthesis. In the culture supernatants, in addition to known calcimycin analogs, eight so far unknown analogs were detected using LC-MS/MS. Under most conditions cezomycin was the compound produced in highest amounts. The highest production of calcimycin was detected upon feeding with glutamine. Supplementation of the medium with glutamic acid resulted in a decrease in calcimycin production, and supplementation of other amino acids such as tryptophan, lysine, and valine resulted in the decrease in the synthesis of calcimycin and of the known intermediates of the biosynthetic pathway. We demonstrated that the production of calcimycin and its analogs is strongly dependent on amino acid supply. Utilization of amino acids as precursors and as nitrogen sources seem to critically influence calcimycin synthesis. Even amino acids not serving as direct precursors resulted in a different product profile regarding the stoichiometry of calcimycin analogs. Only slight changes in cultivation conditions can lead to major changes in the metabolic output, which highlights the hidden potential of biosynthetic gene clusters. We emphasize the need to further study the extent of this potential to understand the ecological role of metabolite diversity originating from single biosynthetic gene clusters.

## 1. Introduction

The polyether ionophore calcimycin (A23187) produced by *S. chartreusis* NRRL 3882 acts as antibiotic and is a well-known biochemical tool with broad use in pharmacological and toxicological studies [1]. Since calcimycin transports divalent cations efficiently across membranes it is used to artificially increase intracellular calcium levels for analysis of calcium-dependent signaling in eukaryotic cells (see [2] for a recent example). Thereby, calcimycin is able to uncouple the oxidative phosphorylation, inhibit ATPase activity, and induce apoptosis in cells [3,4,5]. In the Gram-positive bacterium *Bacillus subtilis* disturbance of the metal ion homeostasis was observed after treatment with calcimycin [6].

**Figure 1:**
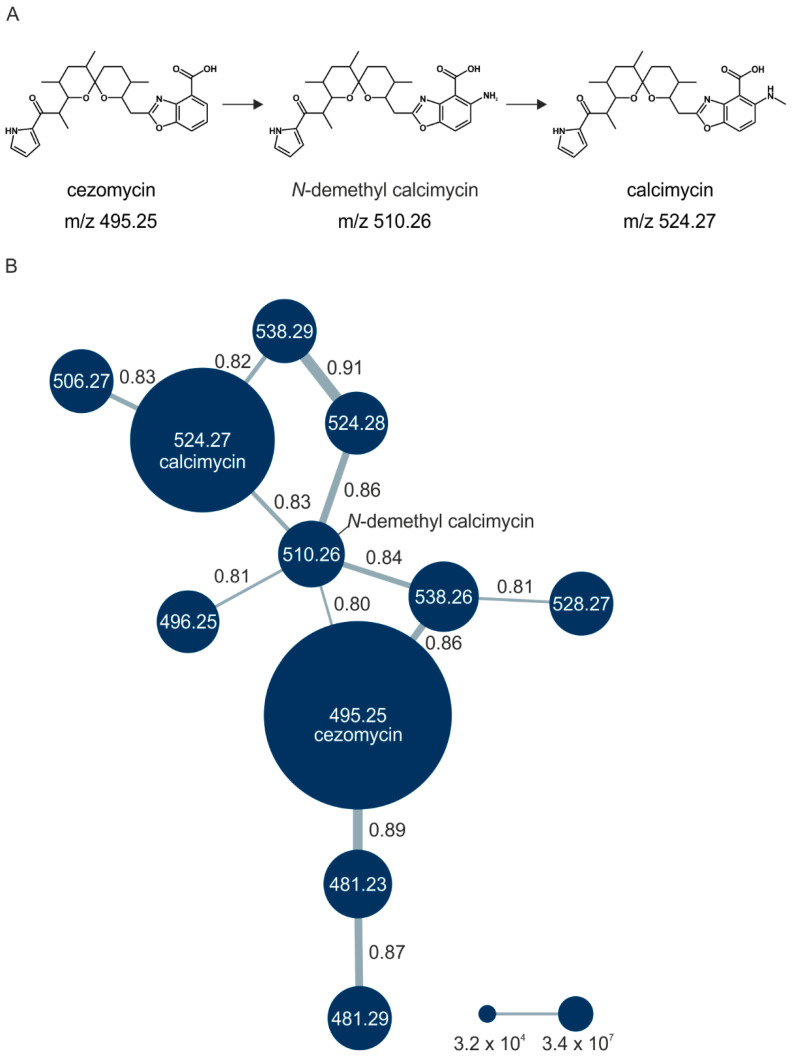
(**A**) Molecular structures of cezomycin, *N*-demethyl calcimycin and calcimycin are shown in successive steps of their biosynthesis in *S. chartreusis*. (**B**) Molecular network of calcimycin analogs. All calcimycin-like metabolites measured in the supernatants of 20 media, each supplemented with one of the proteinogenic amino acids, were clustered according to their cosine similarity calculated from their fragmentation patterns. Analogs with a similarity score of 0.7 or higher were clustered together by a connecting line. The line thickness indicates the degree of structural similarity of the analogs, with the score given above/beside the line. Each node in the cluster resembles one molecule, the measured mass [M + H]^+^ is given with two decimals places here (Table 2 provides all four decimal places). Node size indicates the analogs’ abundance. Small nodes equal 3.2 × 10^4^, largest node equals 3.4 × 10^7^. Abundance was calculated based on area under the curve summed across all 20 growth conditions. Calcimycin: [M + H]^+^ 524.27, cezomycin: [M + H]^+^ 495.25, *N*-demethyl calcimycin: [M + H]^+^ 510.26, others: structurally unknown. All experiments were performed in biological triplicates, except the cultivation with glutamine supplementation, which was done in duplicate.

The biosynthetic pathway of calcimycin is not yet fully understood. Calcimycin is composed of a pyrrole, a polyketide, and a benzoxazole moiety, and this complex structure is the product of a sophisticated multistep biosynthesis [7]. Pyrrole derived from a proline serves as a starter unit for the polyketide synthase [8,9]. A multi-domain enzyme then synthesizes a polyketide chain from propionate and acetate. This chain reacts with 3-hydroxyanthranilic acid, derived from glucose via a shikimate-type pathway, in a hitherto unknown manner to yield the benzoxazole ring system. The emerging intermediate cezomycin is aminated at the benzoxazole moiety to give *N*-demethyl calcimycin, which is further methylated at the amine, leading to the formation of calcimycin (Figure 1A) [7,10]. Besides calcimycin, *S. chartreusis* produces structurally similar analogs utilizing the same biosynthetic pathway [11,12]. The intermediates cezomycin and *N*-demethyl calcimycin possess close structural resemblance to calcimycin with minor variations in substituents at the benzoxazole moiety [10,13]. Other calcimycin analogs differ more strongly in the benzoxazole ring system. Among those is deoxacalcimycin, which possesses a 3-hydroxyanthranilic acid moiety instead of the benzoxazole ring system present in calcimycin [11].

Little is known about the formation and ecological role of calcimycin and its analogs. Previous studies described the involvement of the benzoxazole moiety in ion binding. It was shown by crystallography that the carboxylate oxygen atom and the ring nitrogen atom of benzoxazole together with the pyrrole oxygen play a crucial role in the binding of the calcium ion [14]. Cezomycin, lacking the methylamino group at the benzoxazole ring system, has a ten-times lower binding affinity to calcium than calcimycin [15]. The semisynthetic 4-Br-calcimycin, which possesses a bromide at the benzoxazole ring, in vitro transports calcium into phospholipid vesicles with significantly lower efficiency than calcimycin [16]. These studies show that alterations in the ring system may lead to changes in ion affinities and transport properties.

The production of specialized metabolites and their analogs in streptomycetes often is highly dependent on cultivation conditions [17]. The intermediate of the calcimycin biosynthetic pathway cezomycin was found to be the dominant analog in salt-based chemically defined medium, whereas calcimycin production was not observed under these conditions [11]. However, in complex medium, calcimycin was the main product. The calcimycin analog deoxacalcimycin was detected in complex medium and chemically defined medium supplemented with iron. It was further shown that the inhibitory concentration of calcimycin for *B. subtilis* is highly dependent on the medium composition, especially the concentrations of metal ions [6].

The screening of cultivation conditions is a method frequently used to analyze specialized metabolite production. The alteration of cultivation conditions can lead to high variations in metabolic profiles and the discovery of new specialized metabolites in streptomycetes [11,17,18]. To profile metabolomes of streptomycetes and to find new specialized metabolites, molecular networking has emerged as a valuable tool [11,18,19]. Molecular networks enable the visualization of complex metabolomic datasets by clustering structurally related metabolites [20]. This in particular facilitates the discovery of novel analogs [21]. Directed screening of media components in combination with metabolic networking helps to exploit the potential of single biosynthetic gene clusters (BGC) [11,22].

In this study calcimycin analogs production was systematically analyzed under defined conditions. We presume that specific calcimycin analogs might be preferentially produced under certain conditions to aid in adapting to different environmental conditions. Amino acids are important nitrogen and carbon sources. Some amino acids are direct precursors in calcimycin biosynthesis—proline is needed for the pyrrole moiety, methionine serves as methyl donor for the benzoxazole moiety. In a first attempt to unravel the influence of amino acids on the synthesis of calcimycin and its analogs, *S. chartreusis* was cultivated in chemically defined medium with all canonical amino acids supplemented individually. The produced metabolites were extracted from the culture supernatants and analyzed by LC-MS/MS to assess the biosynthetic output of the calcimycin biosynthetic gene cluster.

## 2. Materials and Methods

### 2.1. Strains and Cultivation Conditions

*S. chartreusis* NRRL 3882 was cultivated in chemically defined medium [21 mM NaCl (Carl Roth, Karlsruhe, Germany), 15 mM (NH_4_)_2_SO_4_ (Sigma-Aldrich, St. Louis, MO, USA), 8 mM MgSO_4_ (VWR International, Darmstadt, Germany), 27 mM KCl (Honeywell International, Morristown, NJ, USA), 50 mM Tris (Sigma-Aldrich, St. Louis, MO, USA), 0.6 mM KH_2_PO_4_ (VWR International, Darmstadt, Germany), 2 mM CaCl_2_ (Avantor, Radnor, PA, USA), 0.01 mM MnSO_4_ (Honeywell International, Morristown, NJ, USA), 11 mM D-glucose (Thermo Fisher Scientific, Waltham, MA, USA), pH 7.5]. The pH was adjusted to 7.5 with HCl. Amino acids were supplemented at a concentration of 0.78 mM. Glutamic acid was added as mono sodium salt. Cultures were incubated for two weeks at 30 °C and 180 rpm in an Innova orbital shaker. If not indicated otherwise, all experiments were conducted three times independently.

### 2.2. Compound Extraction

After the two-week cultivation, 400 µL of the culture supernatant were harvested and extracted with 1320 µL ethyl acetate. Organic and aqueous phases were separated and then washed twice with 200 µL water or 200 µL ethyl acetate, respectively. The phases were dried in vacuo and reconstituted in 100 µL methanol. All reagents used were MS grade.

### 2.3. LC-MS/MS Measurements

The samples were separated with a nanoACQUITY-UPLC system (Waters) with a Mixer Assy (Waters, zirc bead, inner cross Section 1 mm, length 50 mm) and subsequently with an AcquityUPLC HSS T3 column (Waters, pore size 100 Å, particle size 1.8 µm, inner cross section dimension 1 mm, length 100 mm) with a gradient of H_2_O/acetonitrile (ACN) with 0.1% formic acid (FA). The flow rate was 25 µL/min (Table 1).

Masses were recorded in positive resolution mode on a Synapt G2-S HDMS (Waters) with an ESI source and ToF detector. The mass range of 50 to 3000 *m*/*z* was measured with 0.5 s per scan and as a reference mass, leucine encephalin was injected in intervals of 30 s. Parameters used in the measurement: lockspray capillary voltage 2.5 kV, capillary voltage 2.5 kV, cone voltage 30 V, source temperature 120 °C, cone gas flow 60 L/h, flushing gas flow 550 L/h, with a temperature of 150 °C. By collision-induced dissociation (CID) with argon and a collision energy of 10–25 V, fragments were generated. Mass intensity had to exceed 6000 counts/s to start fragmentation for maximum of 6 s and was stopped prematurely if the intensity dropped below the threshold of 6000 counts/s. For spectral analysis MassLynx V4.1 SCN932 (Waters) was used.

### 2.4. Molecular Networking

Files were converted from raw format to mzXML with Proteowizard (version 3.0.9490), with 32-bit binary encoding precision and peak picking for upload onto the Global Natural Products Social Molecular Networking platform (gnps.ucsd.edu) [20] to create a molecular network based on fragmentation spectra. The METABOLOMICS_SNETS 18 workflow was used with the following parameters: parent mass tolerance 2 Da, ion tolerance 0.5 Da, minimal pairs cos 0.7, network topK 10, maximum connected component size 100, minimum matched peaks 6, minimum cluster size 2, run MSCluster. Each node represents a molecule. Molecules with highly similar fragmentation spectra are connected with edges and clustered to a subnetwork. The generated network was visualized in Cytoscape (version 3.6.0) [23] and processed by manual dereplication. Calcimycin and known analogs were annotated by comparing masses and fragmentation spectra with those of previous studies [11].

### 2.5. Heatmap Modeling

Background signals were subtracted from peak signals. A minimum peak area of 100 in at least two out of three replicates was used as cut-off for feature inclusion. Peak areas of features not fulfilling this criterion were recorded as nil. The means of the peak areas across all replicates were calculated and a color gradient was generated for display in a heatmap. Blue indicates a high area value and white an area of 100 or below cut-off.

## 3. Results

### 3.1. Amino Acid Supplementation Leads to the Detection of New Calcimycin Analogs

To analyze the influence of the medium composition on the biosynthesis of calcimycin and its analogs we conducted a cultivation experiment in which *S. chartreusis* was grown in chemically defined medium supplemented with the different proteinogenic amino acids. The culture supernatants were analyzed by LC-MS/MS and to map the calcimycin analogs and measure their production, a molecular network was generated (Figure 1). Using molecular networking, all metabolites from all supernatants were clustered based on similarity of their fragmentation patterns.

Across all 20 media, calcimycin ([M + H]^+^ of 524.2776) was produced along with ten analogs, which were identified by comparing masses, retention times, and fragment spectra (Table 2). For each analog, measured masses and fragment spectra were taken from samples in which they showed the highest abundance. Fragment spectra were compared to a fragment spectrum of calcimycin and corresponding fragments are depicted in black. Besides cezomycin ([M + H]^+^ of 495.2509) and *N*-demethyl calcimycin ([M + H]^+^ of 510.2662), which are structurally known and have been described as intermediates of the biosynthetic pathway, eight new analogs with hitherto unknown structure were detected. Deoxacalcimycin was not detected under any condition tested here. Among the novel calcimycin analogs were the analog with an [M + H]^+^ of 538.26, which had the highest similarities to cezomycin (cosine = 0.86) and *N*-demethyl calcimycin (cosine = 0.84). The analog with an [M + H]^+^ of 481.2369 was structurally most closely related to cezomycin (cosine = 0.89). New analogs similar to calcimycin were the analogs with an [M + H]^+^ of 506.2727 (cosine = 0.83) and an [M + H]^+^ of 538.2975 (cosine = 0.82). The new analogs with the highest structural similarity to *N*-demethyl calcimycin were the analogs with an [M + H]^+^ of 496.2529 (cosine = 0.81) and an [M + H]^+^ of 524.2822 (cosine = 0.86). Two additional new analogs with an [M + H]^+^ of 528.2720 and an [M + H]^+^ of 481.2950 showed similarity to other hitherto unknown analogs. Next, calcimycin and its ten analogs were analyzed regarding their abundance in the culture supernatants. 

### 3.2. Cezomycin Is the Most Abundant Analog in Chemically Defined Medium

**Figure 2:**
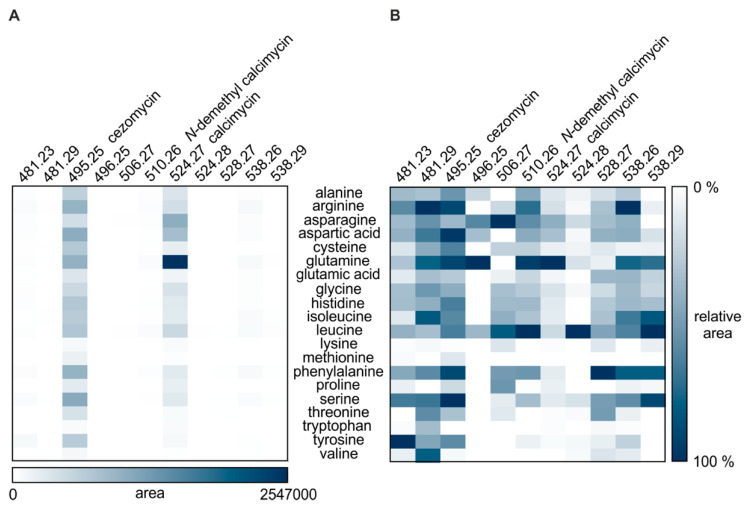
Relative abundance of calcimycin and analogs in culture supernatants of *S. chartreusis* cultivated in chemically defined medium supplemented with amino acids. (**A**) Mean abundance of each analog is displayed in relation to the highest area value determined in the entire calcimycin metabolic network. (**B**) Mean abundance of each analog is displayed in relation to the highest area value measured for the given analog across all cultivation conditions. Metabolites were extracted from the culture supernatants and analyzed by LC-MS/MS. Abundance was calculated based on area under the curve. Background signal was subtracted and mean values were calculated from the replicates. The relative abundance of an analog is displayed in a gradient of white to blue, with blue representing high abundance and white representing absence (cut-off level). All experiments were performed in biological triplicates, except the experiment with glutamine, which was performed in duplicate. Masses are shown with two decimal places (see Table 2 for all four decimal places). Calcimycin: [M + H]^+^ 524.27, cezomycin: [M + H]^+^ 495.25, *N*-demethyl calcimycin: [M + H]^+^ 510.26, others: structurally unknown.

The influence of amino acids on the production of calcimycin and its analogs was visualized in two heatmaps. To gain a global overview of analog abundance, the abundance of each analog was calculated and set in relation to the most abundant metabolite of the entire calcimycin metabolic network (Figure 2A). A second heatmap was generated to aid in assessing for each analog under which condition it was produced most strongly. To this end the abundance in each condition was set in relation to the abundance in the sample in which the analog was most abundant (Figure 2B).

The production of calcimycin and analogs varied with media composition (Figure 2A). Overall, calcimycin and cezomycin were the main metabolites produced from the biosynthetic gene cluster. Supplementation of glutamine to the medium caused the highest accumulation of calcimycin in any culture supernatant. Addition of asparagine and aspartic acid also spurred calcimycin production resulting, however, in less than half the levels produced with glutamine. Glutamine and asparagine were the only amino acids, which led to higher signals for calcimycin than cezomycin. The presence of other amino acids such as lysine, threonine, tryptophan, tyrosine, or valine resulted in lower calcimycin levels. Cezomycin showed the highest abundance in most samples and was detected under all conditions tested. When *S. chartreusis* was cultivated in medium with arginine, aspartic acid, glutamine, phenylalanine, or serine, cezomycin production was high. In contrast, the cultivation in medium with lysine, tryptophan, or valine suppressed the production of cezomycin. The strongest production of *N*-demethyl calcimycin was observed when the medium was supplemented with leucine or glutamine (Figure 2B). As for calcimycin and cezomycin, *N*-demethyl calcimycin production was suppressed by the presence of lysine, tryptophan, and valine. These amino acids resulted in an overall decrease in metabolite synthesis from this biosynthetic gene cluster. In addition, methionine and proline led to a decrease in *N*-demethyl calcimycin synthesis. Beside the main products, which are end products or intermediates of the calcimycin biosynthetic pathway, new calcimycin analogs were detected in supernatants of *S. chartreusis* cultures supplemented with particular amino acids.

### 3.3. The Presence of Canonical Amino Acids Results in the Production of New Analogs

Supplying different amino acids resulted in different amounts of analogs produced (Figure 2B). The newly detected analog with an [M + H]^+^ of 538.2606 was produced in highest amounts in arginine-supplemented medium and was also produced in the presence of glutamine and phenylalanine. Leucine and serine caused an increased production of the analog with an [M + H]^+^ of 538.2975. Addition of tyrosine to the medium gave the highest levels of the analog with an [M + H]^+^ of 481.2369, while arginine caused the highest production of the analog with an [M + H]^+^ of 481.2950. The strongest synthesis of the analog with an [M + H]^+^ of 506.2727 was observed upon addition of asparagine or leucine. While the analog with an [M + H]^+^ of 528.2720 was most abundant in phenylalanine-supplemented medium and the analog with an [M + H]^+^ of 496.2529 when glutamine was added.

## 4. Discussion

In densely populated habitats, survival and growth of bacteria requires the competitive securing of nutrients. Efficient uptake and processing of nutrients are key factors as are strategies to inhibit growth of competitors. The ionophore calcimycin hinders bacterial growth by disrupting the metal ion homeostasis, which was shown using the model soil organism *B. subtilis* [6]. Upon calcimycin treatment, calcium accumulated in the cytosol whereas intracellular manganese and iron levels decreased. Calcimycin analogs seem to transport metal ions with varying efficiencies. The semisynthetic 4-Br-calcimycin is highly efficient in transporting zinc and manganese into phospholipid vesicles in vitro, but much less efficient in transporting calcium [16]. It remains to be investigated whether the natural analogs differ in their transport properties and whether they aid *S. chartreusis* in temporarily adapting to local metal ion concentrations.

Amino acids are attractive nutrients as they act as carbon, nitrogen, and energy sources. They further serve as precursors not only for protein biosynthesis, but also for primary and specialized metabolites. Valine, e.g., is important as precursor of fatty acids for the biosynthesis of macrolides in some *Streptomyces* species [24] and asparagine serves as precursor for clavulanic acid in *Streptomyces clavuligerus* [25]. In fungi, lysine indirectly regulates penicillin biosynthesis by inhibiting homocitrate synthase, the first enzyme of the lysine biosynthesis pathway. This homocitrate synthase catalyzes the formation of α-aminoadipic acid, which is the branch point of the pathways with lysine and penicillin as end products [26,27]. Coming back to calcimycin, amino acids also influence the formation of important building blocks. It was shown that the pyrrole moiety of calcimycin stems from proline [8]. In an early study it was observed that addition of tryptophan to the medium led to an inhibition of calcimycin and *N*-demethyl calcimycin production [9]. Our results revealed that in the presence of tryptophan all but the analog with an [M + H]^+^ of 481.2950 were produced at very low levels (Figure 2B). Isotope-labeling experiments had revealed that tryptophan is not being incorporated into calcimycin [8]. Being eliminated as a direct precursor, the role of tryptophan seems to be a regulatory one. Recent studies have shown the similarity of the proteins CalB1 to CalB4 of the calcimycin biosynthetic gene cluster to enzymes of the phenazine biosynthetic pathway, which can serve as biosynthetic pathway for 3-hydroxyanthranilic acid [28], which is a precursor of the benzoxazole moiety [8]. In phenazine synthesis, 3-hydroxyanthranilic acid is derived from chorismate, synthesized via a shikimate-type pathway. The enzyme CalB4 is annotated as a 3-deoxy-D-arabino-heptulosonate 7-phosphate (DAHP) synthase [7,28], which catalyzes the first step in this pathway. Besides acting as precursor for 3-hydroxyanthranilic acid, chorismate is the precursor of phenylalanine, tryptophan, and tyrosine [29]. Amino acid synthesis is feedback-regulated by its own products [30]. When primary and specialized metabolite biosynthesis utilize the same precursor, the feedback regulation can impact the production of specialized metabolites. For DAHP synthase from *Streptomyces coelicolor* A3(2) and *Streptomyces rimosus* it has already been described that they are inhibited by tryptophan [31]. This mechanism of regulation is likely at play here as well, with tryptophan inhibiting the shikimate pathway, thereby inhibiting cezomycin and calcimycin production. Since tyrosine and phenylalanine are also products of the same chorismate/shikimate biosynthetic pathway, one might have expected them to inhibit calcimycin and analog synthesis as well. However, the synthesis of analogs, including cezomycin is not inhibited by tyrosine or phenylalanine (Figure 2A,B). Many bacteria (including *E. coli*) are known to possess more than one DAHP synthase, which are specific for each end-product [32,33]. This ensures that the pathway is not inhibited completely by the presence of only one of the end products. However, in *Streptomyces* sp. NTK 937 all three synthases were repressed only by one of the final products, namely tryptophan [34]. The genome of *S. chartreusis* also encodes for three DAHP synthases: Basic Local Alignment Search Tool (BLAST) search resulted in detection of two proteins with homologies to CalB4, both of which reveal similarities to DAHP synthases. It is conceivable that as in *Streptomyces* sp. NTK 937, all three synthases are solely repressed by tryptophan, since the other amino acids did not result in a strong repression of calcimycin and analogs’ synthesis (Figure 2A,B). In addition to DAHPs, tryptophan is known to repress anthranilate synthases, which catalyze the conversion of chorismate to anthranilic acid, which then serves as precursor for tryptophan. The protein CalB1 is annotated as an anthranilate synthase and catalyzes the conversion of chorismate to the first product of 3-hydroxyanthranilic acid synthesis, 2-amino 2-desoxyisochorismate (ADIC) [7,28]. This dual inhibition of the same biosynthetic pathway, the repression of the DAHP synthase which limits chorismate availability, and the repression of the anthranilate synthase which causes ADIC depletion, may explain the reduced production of calcimycin and analogs in tryptophan-supplemented medium (Figure 2A,B).

Glutamine is known to serve as a nitrogen donor for purines and pyrimidines as well as nitrogen-containing metabolites. In the first step of the benzoxazole moiety synthesis, CalB1 utilizes chorismate and glutamine to synthesize ADIC [28]. The addition of glutamine to the medium led to the highest production of calcimycin in this study (Figure 2A). The supply of glutamine might lead to an increased synthesis of benzoxazole, which in turn enhances calcimycin production. During later steps of calcimycin biosynthesis, nitrogen is needed to form the amine at the benzoxazole ring system of *N*-demethyl calcimycin [35]. It is conceivable that in this step glutamine serves as a nitrogen donor, tipping the stoichiometry of analogs produced towards calcimycin. Like glutamine, asparagine favored the production of calcimycin over cezomycin (Figure 2A), while glutamic acid, which also is a common nitrogen donor in biosynthetic reactions [36], e.g., in amino acid biosynthesis, did not lead to an enhanced production of calcimycin (Figure 2A). Bacteria are able to utilize diverse nitrogen sources such as ammonia, glutamine, or asparagine. However, not all nitrogen sources are equally conducive to the production of all specialized metabolites. While ammonia favors the production of avilamycin in *Streptomyces viridochromogenus* and avermectins in *Streptomyces avermitilis* [37], it lowers spiramycin biosynthesis in *Streptomyces ambofaciens* [38]. In *Streptomyces cattleya* addition of asparagine to the cultivation medium increased cephamycin C production [39]. Little is known about the mechanisms underlying the positive and negative effects of amino acids and the effect can vary depending on amino acid concentrations and from species to species. It was therefore difficult to predict the influence of amino acids on calcimycin production. The supplementation experiment shows that amino acids do have a strong modulatory effect on the product output of the calcimycin biosynthetic gene cluster.

In the last step of calcimycin biosynthesis, *N*-demethyl calcimycin is transformed to calcimycin by N-methylation [10]. This step is catalyzed by the N-methyltransferase CalM. It has been described that S-adenosyl-L-methionine (SAM), a classical methyl donor, provides the amine for cezomycin methylation [8,10]. One might thus expect that calcimycin production increases upon addition of methionine to the medium. However, the production of calcimycin and analogs on the whole was decreased in medium supplemented with methionine, and calcimycin levels in particular were very low (Figure 2A,B). Increasing synthesis of SAM synthetase or the addition of SAM was described to enhance the production of many antibiotics in streptomycetes [40,41]. To our knowledge it has not been tested whether SAM supplementation also increases production of calcimycin. Since the last biosynthetic step in SAM synthesis from methionine catalyzed by the SAM synthetase is ATP-dependent [42], it might become a rate limiting step when methionine is supplemented instead of SAM. Several other methyl donors can be used for methylation of natural products [43,44]. Thus, the final methylation step in calcimycin biosynthesis might not critically depend on methionine so that earlier biosynthetic steps such as those requiring glutamine are more important factors with regard to determining the biosynthetic output of the calcimycin biosynthetic pathway.

Using isotope labeling a previous study showed that some amino acids like methionine and proline are likely building blocks in calcimycin synthesis [8]. While the present study revealed a strong influence of amino acid availability on the production of calcimycin and analogs, further isotope-labeling experiments need to be conducted to unravel which amino acids act as precursors and which merely trigger different physiological responses.

LC-MS/MS-based metabolomics allows the detection of low abundant compounds. Future efforts will be directed at utilizing the LC-MS/MS data for structure predictions of the analogs. However, to determine the structures and to characterize the biological functions will require their purification, which may be limited by the yield. Media optimization might be needed to further enhance the production of specific calcimycin analogs.

Some so-called silent BGCs have only been found to be active under certain cultivation conditions [45]. This study indicates that this is true not only for the activity of an entire BGC, but also for subsets of a biosynthetic pathway that lead to specific metabolite analogs. This study should encourage us to test different cultivation conditions not only to activate silent BGCs, but also to fully exploit the chemical diversity originating from BGCs.

## 5. Conclusions

The present study was aimed at systematically investigating the influence of amino acid supply on the output of the calcimycin biosynthetic cluster in *S. chartreusis*. In chemically defined medium, the addition of different amino acids led to the production of eight new putative calcimycin analogs. This highlights the diversity of metabolites originating from a single BGC. The ratio of analogs produced varied depending on the amino acid supplemented. Each amino acid fed gave a different output profile of calcimycin analogs, regardless of whether it was a postulated precursor. While tryptophan inhibited the production of calcimycin and analogs, glutamine, likely as precursor of 3-hydroxyanthranilic acid and as nitrogen source, led to high calcimycin levels. This study emphasizes that slight changes in cultivation conditions can have a huge impact on the metabolic output of a BGC. Future studies will be directed at structure elucidation of the analogs, understanding the regulatory mechanisms governing calcimycin and analog production in *S. chartreusis* and at elucidating the biological function of calcimycin and analogs of this polyether ionophore.

## Figures and Tables

**Table 1:** Gradient used for LC-MS/MS analysis.

Time [Min]	% H_2_O with 0.1% FA	% ACN with 0.1% FA
0	95	5
2	95	5
21	0.5	99.5
23	0.5	99.5
28	95	5
30	95	5

**Table 2:** Retention times and fragment spectra of calcimycin analogs detected by LC-MS/MS.

Measured Mass [M + H]^+^/ Δppm ^1^	RT [min]/SD ^2^	Fragment Spectrum ^3^
481.2369/ -	21.39/ 0.19	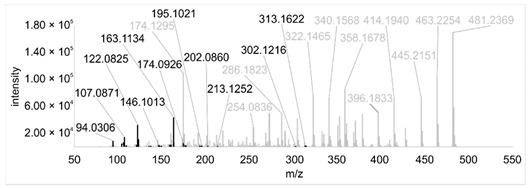
481.2950/ -	19.19/ 3.10	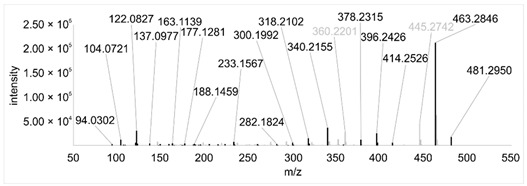
Cezomycin 495.2509/ 2.827	22.83/ 0.18	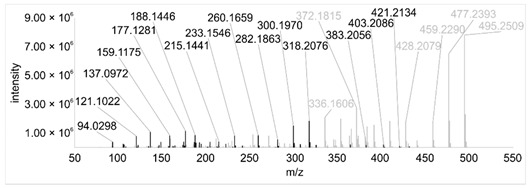
496.2529/ -	22.52/ 0.16	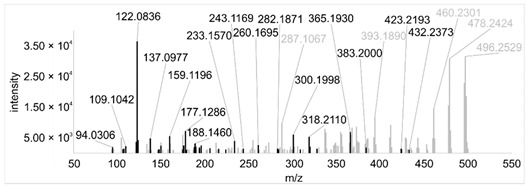
506.2727/ -	24.57/ 0.05	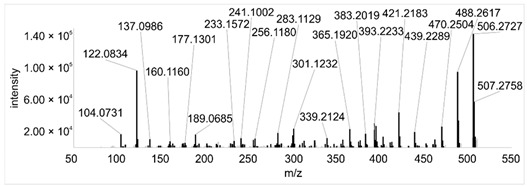
*N*-demethyl- calcimycin 510.2662/ 11.367	23.43/ 0.19	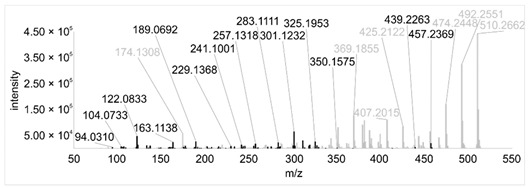
calcimycin 524.2776/ 2.861	24.32/ 0.50	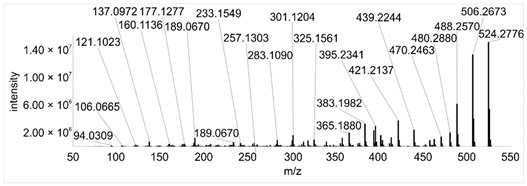
524.2822/ -	23.51/ 0.03	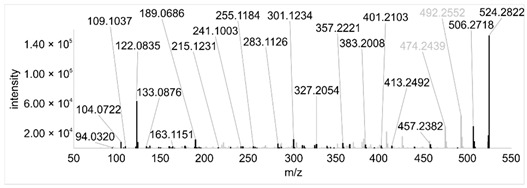
528.2720/ -	19.46/ 0.08	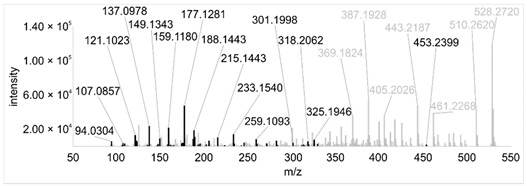
538.2606/ -	23.13/ 0.04	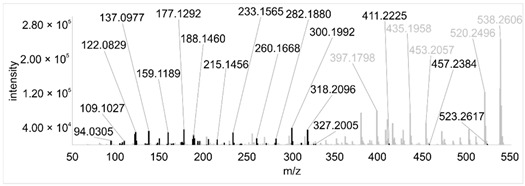
538.2975/ -	24.30/ 0.03	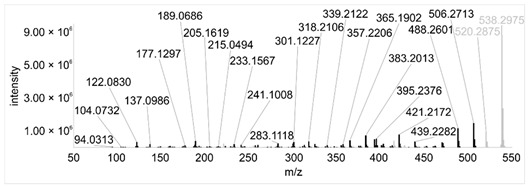

^1^ Representative mass measured in the sample with highest abundance. Deviation of measured mass to theoretical mass in ppm; ^2^ RT: mean retention time, SD: standard deviation; ^3^ Fragment spectra obtained from representative sample with highest abundance. Fragments, which correspond directly to fragments of calcimycin, are highlighted in black.

## Data Availability

The data presented in this study are openly available in the Global Natural Products Social Molecular Networking (GNPS) Library at [doi:10.25345/C5CC0Q] and [http://massive.ucsd.edu/ProteoSAFe/status.jsp?task=aac0abb580e6495fa85c94ff44813a91, accessed on 3 May 2021].

## References

[1] Boot J.H., van Hilten J.A. (1996). The use of the divalent calcium-ionophore A23187 as a biochemical tool in pharmacological and in vitro toxicological studies. Cell Struct. Funct..

[2] Wang X., Yamamoto T., Kadowaki M., Yang Y. (2021). Identification of key pathways and gene expression in the activation of mast cells via calcium flux using bioinformatics analysis. Biocell.

[3] Hara H., Kanazawa T. (1986). Selective inhibition by ionophore A23187 of the enzyme isomerization in the catalytic cycle of sarcoplasmic reticulum Ca^2+^—ATPase. J. Biol. Chem..

[4] Andreo C.S., Vallejos R.H. (1974). Uncoupling of photophosphorylation in spinach chloroplasts by the ionophorous antibiotic A23187. FEBS Lett..

[5] Kajitani N., Kobuchi H., Fujita H., Yano H., Fujiwara T., Yasuda T., Utsumi K. (2007). Mechanism of A23187-induced apoptosis in HL-60 cells: Dependency on mitochondrial permeability transition but not on NADPH oxidase. Biosci. Biotechnol. Biochem..

[6] Raatschen N., Wenzel M., Leichert L.I.O., Düchting P., Krämer U., Bandow J.E. (2013). Extracting iron and manganese from bacteria with ionophores—A mechanism against competitors characterized by increased potency in environments low in micronutrients. Proteomics.

[7] Wu Q., Liang J., Lin S., Zhou X., Bai L., Deng Z., Wang Z. (2011). Characterization of the biosynthesis gene cluster for the pyrrole polyether antibiotic calcimycin (A23187) in *Streptomyces chartreusis* NRRL 3882. Antimicrob. Agents Chemother..

[8] Zmijewski M.J. (1980). Biosynthesis of antibiotic A23187. Incorporation of precursors into A23187. J. Antibiot..

[9] David L., Emadzadeh S. (1982). Biosynthesis of the ionophorus antibiotic A23187. J. Antibiot..

[10] Wu Q., Gou L., Lin S., Liang J., Yin J., Zhou X., Bai L., An D., Deng Z., Wang Z. (2013). Characterization of the N-methyltransferase CalM involved in calcimycin biosynthesis by *Streptomyces chartreusis* NRRL 3882. Biochimie.

[11] Senges C.H.R., Al-Dilaimi A., Marchbank D.H., Wibberg D., Winkler A., Haltli B., Nowrousian M., Kalinowski J., Kerr R.G., Bandow J.E. (2018). The secreted metabolome of *Streptomyces chartreusis* and implications for bacterial chemistry. Proc. Natl. Acad. Sci. USA.

[12] Gou L., Wu Q., Lin S., Li X., Liang J., Zhou X., An D., Deng Z., Wang Z. (2013). Mutasynthesis of pyrrole spiroketal compound using calcimycin 3-hydroxy anthranilic acid biosynthetic mutant. Appl. Microbiol. Biotechnol..

[13] David L., Kergomard A. (1982). Production by controlled biosynthesis of a novel ionophore antibiotic, cezomycin (demethylamino A23187). J. Antibiot..

[14] Smith G.D., Duax W.L. (1976). Crystal and molecular structure of the calcium ion complex of A23187. J. Am. Chem. Soc..

[15] Albrecht-Gary A.M., Blanc S., David L., Jeminet G. (1994). Closely related ionophores cezomycin and calcimycin (A 23187): Cooperative formation of the transporting species. Inorg. Chem..

[16] Erdahl W.L., Chapman C.J., Wang E., Taylor R.W., Pfeiffer D.R. (1996). Ionophore 4-BrA23187 Transports Zn^2+^ and Mn^2+^ with High Selectivity Over Ca^2+^. Biochemistry.

[17] Bode H.B., Bethe B., Höfs R., Zeeck A. (2002). Big Effects from Small Changes: Possible Ways to Explore Nature’s Chemical Diversity. ChemBioChem.

[18] Tangerina M.M.P., Furtado L.C., Leite V.M.B., Bauermeister A., Velasco-Alzate K., Jimenez P.C., Garrido L.M., Padilla G., Lopes N.P., Costa-Lotufo L.V. (2020). Metabolomic study of marine *Streptomyces* sp.: Secondary metabolites and the production of potential anticancer compounds. PLoS ONE.

[19] Bauermeister A., Pereira F., Grilo I.R., Godinho C.C., Paulino M., Almeida V., Gobbo-Neto L., Prieto-Davó A., Sobral R.G., Lopes N.P. (2019). Intra-clade metabolomic profiling of MAR4 *Streptomyces* from the Macaronesia Atlantic region reveals a source of anti-biofilm metabolites. Environ. Microbiol..

[20] Wang M., Carver J.J., Phelan V.V., Sanchez L.M., Garg N., Peng Y., Nguyen D.D., Watrous J., Kapono C.A., Luzzatto-Knaan T. (2016). Sharing and community curation of mass spectrometry data with Global Natural Products Social Molecular Networking. Nat. Biotechnol..

[21] Fang Q., Maglangit F., Wu L., Ebel R., Kyeremeh K., Andersen J.H., Annang F., Pérez-Moreno G., Reyes F., Deng H. (2020). Signalling and Bioactive Metabolites from *Streptomyces* sp. RK44. Molecules.

[22] Machushynets N.V., Wu C., Elsayed S.S., Hankemeier T., van Wezel G.P. (2019). Discovery of novel glycerolated quinazolinones from *Streptomyces* sp. MBT27. J. Ind. Microbiol. Biotechnol..

[23] Shannon P., Markiel A., Ozier O., Baliga N.S., Wang J.T., Ramage D., Amin N., Schwikowski B., Ideker T. (2003). Cytoscape: A software environment for integrated models of biomolecular interaction networks. Genome Res..

[24] Tang L., Zhang Y.X., Hutchinson C.R. (1994). Amino acid catabolism and antibiotic synthesis: Valine is a source of precursors for macrolide biosynthesis in *Streptomyces ambofaciens* and *Streptomyces fradiae*. J. Appl. Bacteriol..

[25] Romero J., Liras P., Martin J.F. (1986). Utilization of ornithine and arginine as specific precursors of clavulanic acid. Appl. Environ. Microbiol..

[26] Luengo J.M., Revilla G., Villanueva J.R., Martin J.F. (1979). Lysine regulation of penicillin biosynthesis in low-producing and industrial strains of *Penicillium chrysogenum*. Microbiology.

[27] Masurekar P.S., Demain A.L. (1972). Lysine control of penicillin biosynthesis. Can. J. Microbiol..

[28] Pavlikova M., Kamenik Z., Janata J., Kadlcik S., Kuzma M., Najmanova L. (2018). Novel pathway of 3-hydroxyanthranilic acid formation in limazepine biosynthesis reveals evolutionary relation between phenazines and pyrrolobenzodiazepines. Sci. Rep..

[29] Bentley R. (1990). The shikimate pathway—A metabolic tree with many branches. Crit. Rev. Biochem. Mol. Biol..

[30] Dewick P.M. (1989). The biosynthesis of shikimate metabolites. Nat. Prod. Rep..

[31] Walker G.E., Dubar B., Hunter I.S., Nimmo H.G., Cogginy J.R. (1996). Evidence for a novel class of microbial 3-deoxy-D-arabino-heptulosonate-7-phosphate synthase in *Streptomyces coelicolor* A3(2), *Streptomyces rimosus* and *Neurospora crassa*. Microbiology.

[32] Brown K.D., Doy C.H. (1963). End-product regulation of the general aromatic pathway in *Escherichia coli* W. Biochim. Biophys. Acta.

[33] Smith L.C., Ravel J.M., Lax S.R., Shiver W. (1962). The control of 3-deoxy-D-arabino-heptulosonic acid 7-phosphate synthesis by phenylalanine and tyrosine. J. Biol. Chem..

[34] Losada A.A., Cano-Prieto C., García-Salcedo R., Braña A.F., Méndez C., Salas J.A., Olano C. (2017). Caboxamycin biosynthesis pathway and identification of novel benzoxazoles produced by cross-talk in *Streptomyces* sp. NTK 937. Microb. Biotechnol..

[35] Wu H., Liang J., Gou L., Wu Q., Liang W.J., Zhou X., Bruce I.J., Deng Z., Wang Z. (2018). Recycling of Overactivated Acyls by a Type II Thioesterase during Calcimycin Biosynthesis in *Streptomyces chartreusis* NRRL 3882. Appl. Environ. Microbiol..

[36] Walker M.C., van der Donk W.A. (2016). The many roles of glutamate in metabolism. J. Ind. Microbiol. Biotechnol..

[37] Zhu C., Lu F., He Y., Han Z., Du L. (2007). Regulation of avilamycin biosynthesis in *Streptomyces viridochromogenes*: Effects of glucose, ammonium ion, and inorganic phosphate. Appl. Microbiol. Biotechnol..

[38] Lebrihi A., Lamsaif D., Lefebvre G., Germain P. (1992). Effect of ammonium ions on spiramycin biosynthesis in *Streptomyces ambofaciens*. Appl. Microbiol. Biotechnol..

[39] Khaoua S., Lebrihi A., Germain P., Lefebvre G. (1991). Cephamycin C biosynthesis in *Streptomyces cattleya*: Nitrogen source regulation. Appl. Mircobiol. Biotechnol..

[40] Kim D.J., Huh J.H., Yang Y.Y., Kang C.M., Lee I.H., Hyun C.G., Hong S.K., Suh J.W. (2003). Accumulation of S-adenosyl-L-methionine enhances production of actinorhodin but inhibits sporulation in *Streptomyces lividans* TK23. J. Bacteriol..

[41] Huh J.H., Kim D.J., Zhao X.Q., Li M., Jo Y.Y., Yoon T.M., Shin S.K., Yong J.H., Ryu Y.W., Yang Y.Y. (2004). Widespread activation of antibiotic biosynthesis by S-adenosylmethionine in streptomycetes. FEMS Microbiol. Lett..

[42] Fontecave M., Atta M., Mulliez E. (2004). S-adenosylmethionine: Nothing goes to waste. Trends Biochem. Sci..

[43] Li S.M., Westrich L., Schmidt J., Kuhnt C., Heide L. (2002). Methyltransferase genes in *Streptomyces rishiriensis*: New coumermycin derivatives from gene-inactivation experiments. Microbiology.

[44] Inahashi Y., Zhou S., Bibb M.J., Song L., Al-Bassam M.M., Bibb M.J., Challis G.L. (2017). Watasemycin biosynthesis in *Streptomyces venezuelae*: Thiazoline C-methylation by a type B radical-SAM methylase homologue. Chem. Sci..

[45] Rutledge P.J., Challis G.L. (2015). Discovery of microbial natural products by activation of silent biosynthetic gene clusters. Nat. Rev. Microbiol..

